# Temporal changes in cecal luminal and mucosal microbiota of broiler chickens with clinical coccidiosis (*Eimeria maxima*)

**DOI:** 10.1371/journal.pone.0321212

**Published:** 2025-04-24

**Authors:** Philip M. Campos, Katarzyna B. Miska, Mark C. Jenkins, Monika Proszkowiec-Weglarz

**Affiliations:** 1 Department of Agriculture, Animal Biosciences and Biotechnology Laboratory, Beltsville Agricultural Research Center, Agricultural Research Service, United States, Beltsville, Maryland, United States of America; 2 Department of Agriculture, Animal Parasitic Diseases Laboratory, Beltsville Agricultural Research Center, Agricultural Research Service, United States, Beltsville, Maryland, United States of America; Beni Suef University Faculty of Veterinary Medicine, EGYPT

## Abstract

Coccidiosis is a gastrointestinal disease caused by *Eimeria* parasites which leads to major economic losses in the poultry industry worldwide. *Eimeria* infection may alter the gut microbiota, which has been associated with chicken health and performance. This study aimed to determine the effects of *Eimeria maxima* infection on the luminal and mucosal microbiota of the cecum (CeL and CeM) at multiple time points post-infection (days 3, 5, 7, 10, and 14). Infection decreased Shannon diversity at d 3 (*P* = 0.03), increased observed features (ASVs) at d 5 (*P* < 0.01), and increased Shannon diversity at d 10 (*P* = 0.04) in the CeL microbiota compared to the control. In CeM microbiota, infection increased observed features at d 5 (*P* = 0.03), but later decreased observed features at d 14 (*P* = 0.01). Relative abundance of potential butyrate-producing bacteria such as [*Ruminococcus*] *torques group* in CeL and *Butyricicoccus* in CeM were decreased in infected birds, and some metabolic pathways related to butyrate production were predicted to be decreased. These findings show *E. maxima* may affect cecal microbiota alpha diversity in a time-dependent manner and reduce abundance of bacteria potentially important to gut health.

## Introduction

Coccidiosis is a parasitic disease characterized by infection of the gastrointestinal tract (GIT) by apicomplexan protozoa of the genus *Eimeria*. At least seven species of *Eimeria* are known to infect chickens, with the most studied species in farms being *Eimeria maxima*, *Eimeria acervulina*, *and Eimeria tenella* [1]. These species cause schizogony in the intestinal submucosa with severe destruction of the mucosa, favoring secondary infections and causing morbidity and mortality [2,3]. Damage caused by *Eimeria* may lead to malabsorption, inflammation, and accumulation of mucus, further leading to weight gain depression, inefficient feed conversion, and reduced egg production [4]. Overall, coccidiosis has a significant economic impact on the poultry industry, with annual losses from morbidity, mortality, and costs for anticoccidial drugs and vaccines estimated to total US $14 billion worldwide [5]. Prophylactic drugs in feed and vaccines have been relied upon to control coccidiosis, however, increasing resistance to drugs has spurred research into alternatives to antimicrobials, such as probiotics, prebiotics, antioxidants, essential oils, and feed additives [6,7].

It is important to understand the effectiveness of such alternatives by researching the gut microbiota, or communities of bacteria residing in the GIT. The gut microbiota has been shown to be important for chicken health, being associated with nutrient exchange, immune system modulation, digestive system physiology, and pathogen exclusion [8,9]. Gut microbiota balance can be disturbed by stressors such as infection, with various studies demonstrating effects from *Eimeria* infections in different regions of the GIT, including the cecum, ileum, jejunum, and duodenum [10–15]. Bacterial composition differs in these regions, especially in the cecum where absolute bacterial counts and diversity of bacterial taxa are much higher [16]. The cecal microbiota can contain short-chain fatty acids (SCFA) producers from the Lachnospiraceae and Ruminococcaceae families, which may have beneficial effects through energy production and reduction of pH in the intestine [17], and some taxa have been correlated with body weight gain [12]. Additionally, composition of performance-linked microbes in the cecum has been correlated with improved feed conversion ratio and increased metabolizable energy [17].

Different *Eimeria* species target different regions of the GIT and vary in pathogenicity, affecting the degree of changes to the microbiota during infection. For example, *E. tenella* directly targets the cecum and its high pathogenicity causes a high amount of physical damage [18], which can lead to a temporary dominance of *Escherichia-Shigella* in the cecal microbiota during the peak of infection (7 d post-infection) [12]. In contrast, *E. acervulina*, which targets the duodenum and upper jejunum and has the lowest pathogenicity compared to *E. tenella* and *E. maxima* [18,19], did not have such an effect on *Escherichia-Shigella* abundance in the cecum [15]. However, *E. acervulina* infection still shared some similar effects to that of *E. tenella* despite its weaker pathogenicity and different target region, such as decreasing the relative abundance of potential SCFA-producing bacteria, including members of Lachnospiraceae [15].

As *E. maxima* has moderate pathogenicity in between *E. acervulina* and *E. tenella* and has a predilection for infecting the jejunum [18,19], we hypothesized *E. maxima* infection may alter the cecal microbiota and abundances of some SCFA-producing bacteria in a similar manner to *E. acervulina*, however, without the major change in dominant bacteria seen from *E. tenella* infection. While other studies have shown *E. maxima* infection can alter broiler cecal microbiota diversity and/or composition together with other *Eimeria* species [14], with *Clostridium perfringens* [20], or in an interaction with time post-infection and butyric acid glycerol esters [21], less is known on the individual effects of *E. maxima* infection. This study aimed to determine the temporal changes during a 14-day period on the cecal luminal and mucosal microbiota following infection by *E. maxima*.

## Materials and methods

### Animal care and tissue sampling

All animal care procedures were approved by the Institutional Animal Care and Use Committee (IACUC, protocol #18–025) of the Beltsville Agricultural Research Center (BARC). This study was performed and reported in accordance with ARRIVE guidelines (https://arriveguidelines.org/). Ross 708 male broilers (288 birds, 1 day of age) were obtained from Longnecker’s Hatchery (Elizabethtown, PA) and placed into 1.00 m^2^ open-top wire brooder pens (approximately 25 chicks per pen). At d 19, birds were moved into 72 battery cages (Alternative Designs, Siloam Springs, AR) with 4 birds per pen. A corn-soybean-based diet (approximately 24% crude protein in crumble format) and water were provided to chicks *ad libitum* for the duration of the study. Treatment groups were formed by infecting half of the birds (144 birds) with 1 × 10^3^ *E. maxima* oocysts (USDA APU1 isolate) in a volume of 1.0 mL per bird by oral gavage at d 21 (infected [IF]), while the remaining 144 birds were sham-infected with water (control [C]). Prevention of cross-transmission was achieved by placing the resulting 36 pens of C birds and 36 pens of IF birds in separate areas of the facility, and C birds were checked at sampling time points to ensure no symptoms of accidental infection were present.

At day 0, 3, 5, 7, 10, and 14 post-infection (PI), a chicken closest to the pen’s average weight (calculated from the 4 birds to prevent sampling of birds with outlier weights) was euthanatized via cervical dislocation. There were 6 pens (n = 6 replicates per treatment) for each time point, resulting in 36 C birds and 36 IF birds euthanized. Humane endpoints could not be used in the study design because the alternative of fecal sampling would not be as accurate as directly sampling the GIT of chickens, unlike in other animals such as mice. This is because investigators cannot control for the time the chicken excreta is voided, therefore, attempting to collect samples at one time point would mean samples were exposed to air for different time periods, and this would affect the number of anaerobic bacteria present in the final results [16]. Parameters to evaluate infection were measured at each time point, including plasma carotenoid concentrations, body weight gain, and feed conversion ratio. For luminal (CeL) and mucosal (CeM) microbiota sampling, a total of 60 samples were randomly chosen, including 30 IF samples (n = 6 at five time points from d 3 to d 14 PI) and 30 C samples (n = 5 at 6 time points from d 0 to d 14 PI). One of the paired ceca was dissected to obtain cecal contents for CeL microbiota and cecal epithelial scrapings for CeM microbiota. Isolated specimens were snap frozen in liquid nitrogen and stored at -80 °C until bacterial DNA isolation.

### Library preparation and sequencing

DNA extraction, library preparation, and sequencing were performed as described in Campos et al. (2021), utilizing a DNeasy PowerSoil kit (Qiagen, Valencia, CA), PCR primers targeting the V3-V4 region of the 16S rRNA gene, and the Illumina MiSeq platform (Illumina, Inc., San Diego, CA), respectively. The 16S rRNA gene sequences determined in this study were deposited in the NCBI Sequence Read Archive database (SRA accession no. PRJNA1122195).

### Bioinformatics and data analysis

CeL and CeM microbiota datasets were analyzed using the bioinformatics platform Quantitative Insights Into Microbial Ecology 2 (QIIME 2) version 2023.2 [22]. Quality control and denoising were performed on demultiplexed, paired-end sequence data with DADA2 [23] *via* the q2-dada2 plugin, using a median quality cutoff of 30 to determine truncation settings. Taxonomic classification was initiated by obtaining RESCRIPt pre-formatted SILVA [24] version 138 99% reference sequences and taxonomy files from the QIIME 2 Data Resources page (https://docs.qiime2.org/2023.2/data-resources/). RESCRIPt is a process used to reduce inconsistencies and improve processing by removing duplicate sequences that are assigned different taxonomies [25]. The SILVA database was preferred over the commonly used Greengenes 2013 database as interpretation of results may improve because of its larger size and more recent updates to taxonomy [26,27]. Reads were extracted from reference sequences using the V3-V4 region forward and reverse primers (5’-end: CCTACGGGNGGCWGCAG and 3’-end: GACTACHVGGGTATCTAATCC, respectively) and used to produce a feature classifier *via* q2-feature-classifier fit-classifier-naive-bayes [28]. Next, taxonomy was assigned *via* the q2-feature-classifier classify-sklearn naïve Bayes taxonomy classifier to the amplicon sequence variants (ASVs) produced from DADA2 processing. All ASVs were aligned with MAFFT [29] *via* q2-alignment and used to construct a phylogeny with FastTree2 [30] *via* q2-phylogeny. An alpha rarefaction plot produced *via* q2-diversity was utilized to determine sampling depth where rarefaction, or subsampling without replacement, would maintain most of the bacterial diversity. Sampling depths of 13,067 for CeL and 37,522 for CeM were applied for alpha and beta diversity analyses *via* q2-diversity.

Diversity metrics can indicate whether the microbiota is altered between a control and treatment. Alpha diversity is a measure of species richness and/or evenness within an individual sample was analyzed with four metrics: Shannon diversity index, observed features (ASVs), Faith’s phylogenetic diversity (Faith PD) [31], and evenness. Observed features and Faith PD are measures of richness, with observed features measuring the number of ASVs and Faith PD calculating richness based on phylogenetic differences [31]. Statistical analysis of alpha diversity was performed using the non-parametric Kruskal-Wallis test to compare diversity between IF and C treatments at each time point. Beta diversity is a measure of distance between bacterial compositions of each microbiota sample and was measured using UniFrac distance metrics, which calculate distance based on phylogenetic tree branch lengths [32]. Unweighted UniFrac considers the presence and absence of ASVs in samples, while weighted UniFrac considers the abundance of ASVs [33]. Statistical analysis to determine differences in treatment groups at each time point was performed using the non-parametric permutational analysis of variance (PERMANOVA) test. Distances between differing microbiota and clustering of similar samples were visualized using principal coordinates analysis (PCoA). Visualizations for alpha diversity significance and beta diversity PCoA were produced in R 4.3.2 [34] using the packages QIIME2R 0.99.35 [35] to import QIIME 2 PCoA results and tidyverse 2.0.0 [36] for data wrangling with dplyr and figure production with ggplot2.

The linear discriminant analysis effect size (LEfSe) algorithm was used to analyze differential bacterial abundance between IF and C birds [37]. Feature tables at the genus level were converted to relative abundance, exported from QIIME 2, and analyzed at the Huttenhower Lab Galaxy web server (http://huttenhower.sph.harvard.edu/galaxy) using the default parameters, including a LDA cutoff score of 2.0. Functional abundances were predicted based on marker gene sequences using Phylogenetic Investigation of Communities by Reconstruction of Unobserved States 2 (PICRUSt2) version 2.4.2 [38]. Functional abundance data was produced using the MetaCyc Metabolic Pathways Database [39], and STAMP 2.1.3 [40] was used to analyze and visualize differential abundance of the predicted pathways.

## Results

### Infection with *Eimeria maxima*

Plasma carotenoid concentrations along with decrease in body weight gain (BWG) were used to verify a successful *E. maxima* infection, and the results have been reported previously [41]. Carotenoids were significantly decreased in IF birds during d 7, 10, and 14 compared to C birds and were at their lowest point at d 7. BWG was significantly lower in IF birds overall, with decreases during the same period carotenoids were decreased (d 7, 10, and 14).

### Microbiota profiles

**Fig 1:**
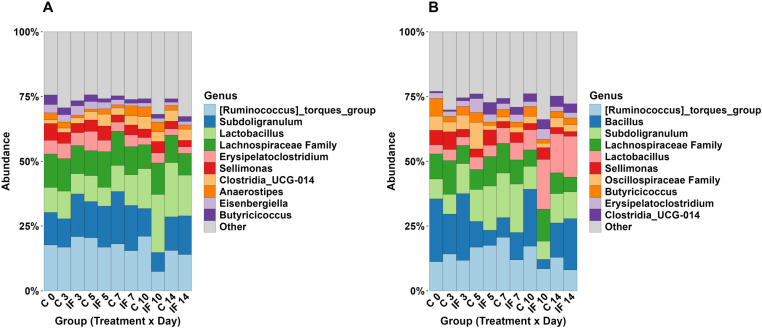
Summary of bacterial composition (relative abundance, %) for samples in each group from **(A)** CeL and **(B)** CeM microbiota at the genus level. In each legend, the 10 most common genera overall are listed.

Sequencing and data processing numbers for the CeL and CeM microbiota datasets are summarized in Table 1, including information on the number of raw reads, the average number of reads after processing, and total ASVs and their average read length. [*Ruminococcus*] *torques group*, *Subdoligranulum*, *Lactobacillus*, unclassified Lachnospiraceae, and *Erysipelatoclostridium* were the five most abundant bacterial taxa at the genus level in CeL microbiota (Fig 1A). In CeM microbiota, [*Ruminococcus*] *torques group*, *Bacillus*, *Subdoligranulum*, unclassified Lachnospiraceae, and *Lactobacillus* were the five most abundant bacterial taxa at the genus level (Fig 1B).

**Table 1:** Sequencing summary of CeL and CeM microbiota datasets processed in QIIME 2. QC = quality control via DADA2, ASVs = amplicon sequence variants.

	CeL	CeM
Number of samples	60	60
Raw reads	6,097,593	14,032,303
Reads after QC	4,670,898	9,680,873
Reads per sample (range)	6,466–206,342	387–547,945
Mean reads per sample	77,848	161,347
Sampling depth	13,067	37,522
Total number of ASVs	682	1,106
ASV read length (range)	282–504	268–472
Mean ASV read length	415	401

### Alpha diversity

**Fig 2:**
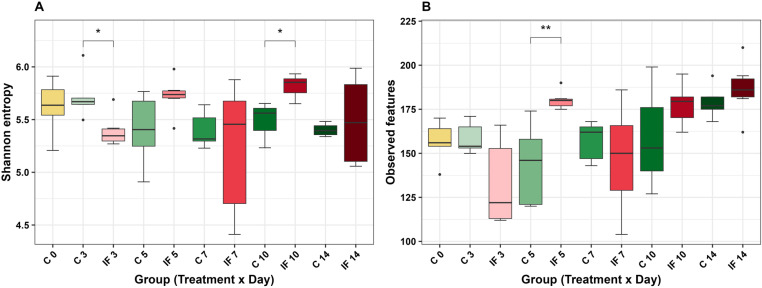
Comparisons of alpha diversity metrics in the cecal luminal microbiota: **(A)** Shannon diversity and (B) observed features (ASVs). Stars (*) denote significant differences (* = *P* < 0.05, ** = *P* < 0.01).

First, significance was evaluated based on group (infection status x time post-infection) overall, then if significant, pairwise comparisons at each time point were used to determine significant differences in IF and C birds. Shannon diversity significantly differed based on group (Kruskal-Wallis, H = 18.55, *P* = 0.046) in CeL microbiota. Infection decreased Shannon diversity on d 3 (H = 4.80, *P* = 0.03, Fig 2A) compared to the C, but later increased Shannon diversity on d 10 (H = 4.27, *P* = 0.04, Fig 2A). The number of observed features (ASVs) significantly differed based on group (H = 31.51, *P* < 0.01), with observed features increasing in IF birds on d 5 compared to C birds (H = 7.53, *P* < 0.01, Fig 2B). Faith PD did not significantly differ based on group (*P* > 0.05). Evenness differed based on group overall (H = 18.61, *P* = 0.046), however, at each time point, evenness did not significantly differ between IF and C birds (all *P* > 0.05).

**Fig 3:**
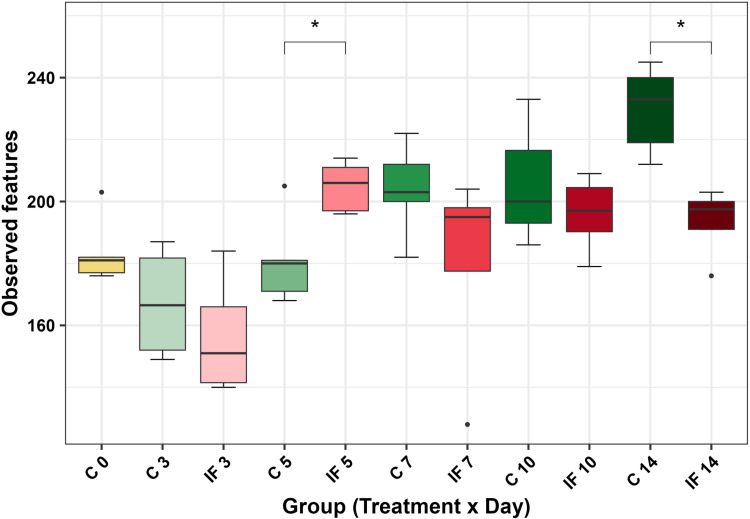
Comparison of the observed features (ASVs) alpha diversity metric in the cecal mucosal microbiota. Stars (*) denote significant differences (* = *P* < 0.05).

The metrics affected and the timing of effects were different in CeM microbiota. Observed features differed based on group (H = 29.71, *P* < 0.01), with infection increasing the number of observed features in IF birds on d 5 (H = 4.81, *P* = 0.03, Fig 3) and d 14 (H = 6.00, *P* = 0.01, Fig 3). The remaining metrics Shannon diversity, Faith PD, and evenness were not significantly different based on group (all *P* > 0.05).

### Beta diversity

**Fig 4:**
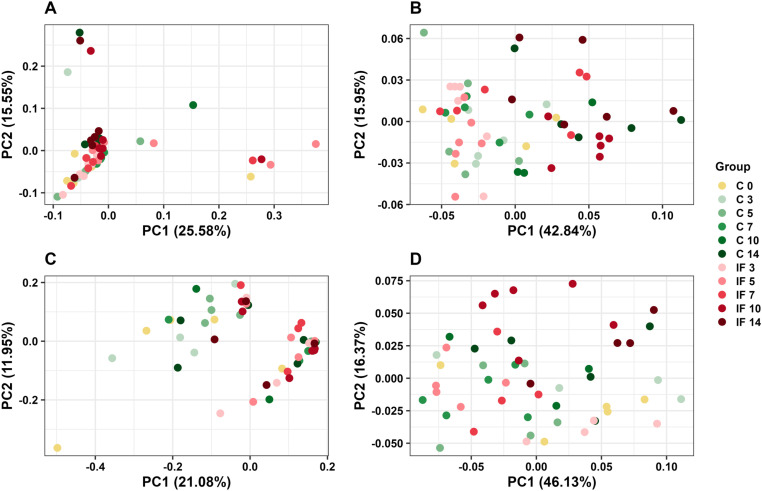
Principal coordinate analysis (PCoA) comparing *Eimeria maxima*-infected birds and control birds at multiple time points based on (A) unweighted UniFrac distance matrix in CeL microbiota, (B) weighted UniFrac distance matrix in CeL microbiota, (C) unweighted UniFrac distance matrix in CeM microbiota, and (D) weighted UniFrac distance matrix in CeM microbiota.

Distances between microbiota profiles were measured with unweighted and weighted UniFrac metrics and used to determine if IF birds had distinct microbiota compared to C birds at each time point. Although the unweighted UniFrac (pseudo-F = 1.86, *P* < 0.01, Fig 4A) and weighted UniFrac (pseudo-F = 3.75, *P* < 0.01, Fig 4B) metrics were significant for groups overall in CeL microbiota, there were no significant differences between IF and C birds at each time point for both metrics (all *P* > 0.05). CeM microbiota followed the same pattern, where both unweighted UniFrac (pseudo-F = 1.53, *P* < 0.01, Fig 4C) and weighted UniFrac (pseudo-F = 2.92, *P* < 0.01, Fig 4D) were significant for groups overall, however, there were no significant differences between IF and C birds at each time point (all *P* > 0.05).

### Differential abundance of bacterial taxa

**Fig 5:**
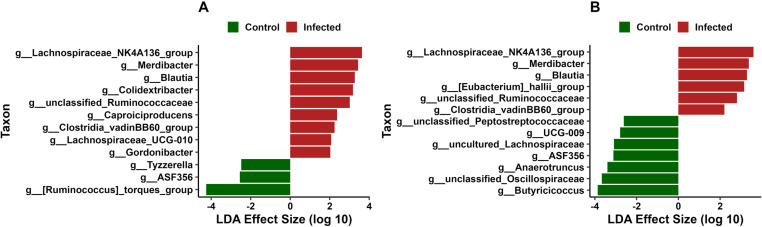
Differential bacterial abundance analysis using linear discriminant analysis effect size (LEfSe) in: (A) cecal luminal and (B) cecal mucosal microbiota. Positive effect size (red bars) indicates higher relative abundance in *Eimeria maxima*-infected birds, while negative effect size (green bars) indicates higher relative abundance in control birds.

CeL microbiota contained nine genera that were in greater relative abundance in IF birds compared to C birds (Fig 5A), including Lachnospiraceae *NK4A136 group* (Effect size [ES] = 3.64, *P* = 0.03), *Merdibacter* (ES = 3.44, *P* = 0.02), *Blautia* (ES = 3.27, *P* = 0.03), *Colidextribacter* (ES = 3.18, *P* = 0.02), and unclassified Ruminococcaceae (ES = 3.02, *P* < 0.01). Those in greater relative abundance in C birds were [*Ruminococcus*] torques group (ES = 4.25, *P* = 0.01), *ASF356* (family Lachnospiraceae, ES = 2.55, *P* = 0.03), and *Tyzzerella* (ES = 2.48, *P* < 0.01) (Fig 5A).

CeM microbiota contained six genera were in greater relative abundance in IF birds (Fig 5B), including Lachnospiraceae *NK4A136 group* (ES = 3.60, *P* = 0.03), *Merdibacter* (ES = 3.37, *P* = 0.03), *Blautia* (ES = 3.29, *P* = 0.02), [*Eubacterium*] *halli group* (ES = 3.15, *P* = 0.03), and unclassified Ruminococcaceae (ES = 2.81, *P* = 0.03). Seven genera were in greater relative abundance in C birds (Fig 5B), including *Butyricicoccus* (ES = 3.86, *P* = 0.02), unclassified Oscillospiraceae (ES = 3.67, *P* = 0.02), *Anaerotruncus* (ES = 3.40, *P* = 0.04), *ASF356* (ES = 3.11, *P* < 0.01), and uncultured Lachnospiraceae (ES = 3.08, *P* < 0.01).

### Predicted functional abundances

**Fig 6:**
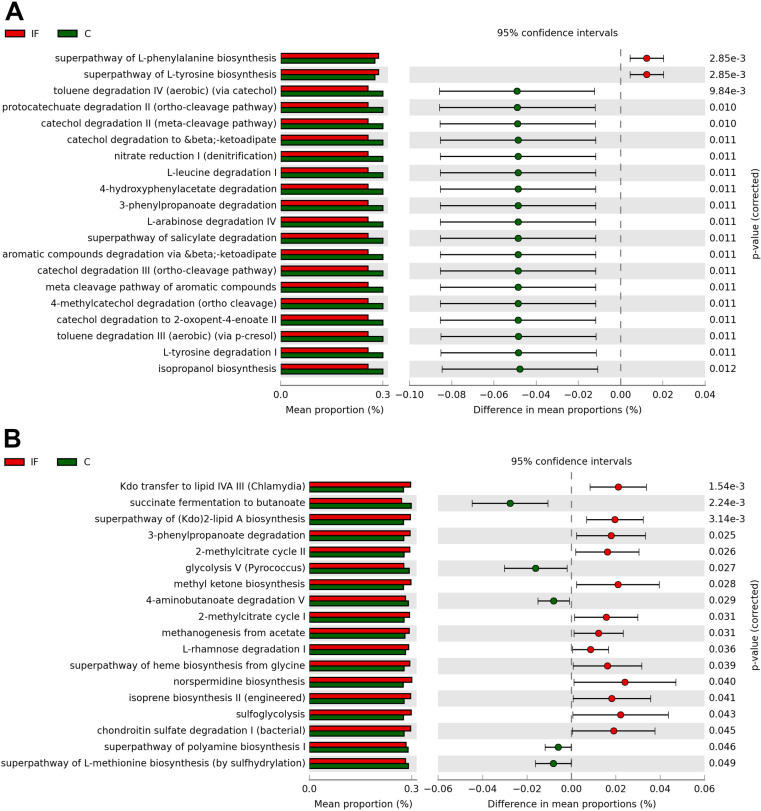
Effect of *Eimeria maxima*-infection on the differential mean proportion (%) of predicted MetaCyc pathways in the (A) cecal luminal and (B) cecal mucosal microbiota.

Genes for the predicted MetaCyc pathways superpathway of L-phenylalanine biosynthesis (*P* < 0.01) and superpathway of L-tyrosine biosynthesis (*P* < 0.01) were in greater relative abundance in the CeL microbiota of IF birds compared to those of C birds (Fig 6A). There were 31 pathways predicted to be in greater relative abundance in C birds (all *P* < 0.05), including toluene degradation IV (aerobic, via catechol) (*P* < 0.01), protocatechuate degradation II (ortho-cleavage) (*P* = 0.01), catechol degradation II (meta-cleavage) (*P* = 0.01), catechol degradation to β-ketoadipate (*P* = 0.01), nitrate reduction I (denitrification) (*P* = 0.01).

Thirteen pathways were predicted in be in greater relative abundance in the CeM microbiota of IF birds (Fig 6B), including Kdo transfer to lipid IVA III (*Chlamydia*) (*P* < 0.01), superpathway of (Kdo)2-lipid A biosynthesis (*P* < 0.01), 3-phenylpropanoate degradation (*P* = 0.03), 2-methylcitrate cycle II (*P* = 0.03), and methyl ketone biosynthesis (*P* = 0.03). The pathways succinate fermentation to butanoate (*P* < 0.01), glycolysis V (*Pyrococcus*) (*P* = 0.03), 4-aminobutanoate degradation V (*P* = 0.03), superpathway of polyamine biosynthesis I (*P* = 0.046), and superpathway of L-methionine biosynthesis (by sulfhydrylation) (*P* = 0.049) were predicted to be in greater relative abundance in C birds.

## Discussion

This study investigated the effects of *Eimeria maxima* infection on bacterial alpha and beta diversity, relative abundances, and predicted metabolic function in cecal luminal and mucosal microbiota during a 14-day post-infection period. *E. maxima* affected alpha diversity as hypothesized, however, neither CeL or CeM microbiota were considered distinct under beta diversity analyses between the IF and C birds at each time point PI. Alpha diversity results showed that infection decreased Shannon diversity at d 3 in CeL microbiota, but later increased Shannon diversity at d 10. This later increase of Shannon diversity at d 10 is different to what was observed from *E. acervulina* infection, where there was a trend of decreasing Shannon diversity (and a significant decrease in evenness) at this point [15]. This may indicate the early decrease at d 3 from *E. maxima* infection influenced how the microbiota changed over time in IF birds. An increase in observed features (ASVs) in IF birds on d 5 suggests that the reduced diversity on d 3 may have provided an opportunity for other bacterial taxa to populate the CeL microbiota. The increase in observed ASVs on d 5 also occurred in the CeM microbiota of IF birds, though observed ASVs then decreased in IF birds at d 14. This illustrates that at one time point, the CeL and CeM microbiota can be affected similarly, but at another time point a different effect may occur. Interestingly, this pattern at d 5 and d 14 was the same in CeM microbiota of *E. acervulina* infected birds, despite the patterns differing in CeL microbiota between the *E. maxima* and *E. acervulina* studies. Similar to previous studies, this result demonstrates the usefulness of measuring both luminal and mucosal microbiota to capture all possible changes from an *Eimeria* infection [12,13,15].

The beta diversity result is somewhat surprising, since infection with the less pathogenic *E. acervulina* had previously been shown to result in distinct CeL and CeM microbiota at certain time points [15]. Although *E. tenella* is the species expected to directly alter the cecal microbiota, it has been discussed that *E. maxima* and *E. acervulina* may indirectly alter the cecal microbiota by affecting proteolytic fermentation in the small intestine and increasing the endogenous loss of proteins [42,43]. One possible explanation is that higher doses of *E. acervulina* (1 x 10^5^ oocysts) are utilized compared to *E. maxima* (1,000 oocysts) in these studies to account that more *E. acervulina* are necessary to observe infection, leading to more overall disturbance to microbiota of IF birds from the number of oocysts. A recent study investigating the effects of *E. maxima* on cecal digesta microbiota utilized more oocysts (2 x 10^4^) during infection and found significant differences between weighted UniFrac profiles of C and IF birds on d 5 and 7 [44]. Another explanation is that variation in the initial microbiota of individual broilers in this study resulted in different responses to infection than in previous studies, possibly from time of year the birds were raised, since the broiler breed and facilities remained the same. This difference in profiles could make the microbiota less susceptible to overall changes in composition. For instance, [*Ruminococcus*] *torques group* (hereafter, *R. torques*) was the dominant bacterium at the genus level in microbiota of this study, while unclassified Lachnospiraceae was the more dominant group in the *E. acervulina* study [15]. Together, the alpha and beta diversity results suggest that *E. maxima* altered diversity within the microbiota of individuals at certain time points but did not cause microbiota of IF and C birds to cluster into separate groups based on overall composition.

Although overall composition was not distinct at a particular time point, differential abundance analysis showed that infection affected relative abundances of some bacterial genera between IF and C birds. In CeL microbiota, the largest effect observed was a decrease in *R. torques* in IF birds. Although *R. torques* was not decreased in CeM microbiota of IF birds, other bacteria potentially playing a similar role as butyrate-producers such as *Butyricicoccus* [45,46] and *Anaerotruncus* [47] were decreased. This result is consistent with the effect observed from *E. acervulina* infection on CeM microbiota, where the family Butyricicoccaceae and *Anaerotruncus* were also decreased in IF birds [15]. Results have also been consistent in the CeL microbiota under our studies on different *Eimeria* species, where generally a member of the Lachnospiraceae family is likely to be the most abundant bacterial taxa, and the abundance of this member is likely to be decreased in birds infected with *Eimeria* [12,13]. This consistency is notable as the pathogenicity and target locations of *E. acervulina*, *maxima*, and *tenella* differ, and only *E. tenella* directly targets the cecum. In this study, the pattern was observed with *R. torques*, rather than an unclassified Lachnospiraceae group as in the *E. acervulina* and *E. tenella* studies, however, *R. torques* also belongs to the Lachnospiraceae family, a family that is known to contain producers of short-chain fatty acids (SCFAs) such as butyrate [48–50]. It is unclear how similar or different *R. torques* is in function compared to the unclassified Lachnospiraceae group. *R. torques* has also been correlated with SCFA concentration [51], but may have other functions as a mucin degrader, in which higher abundance could lead to impaired gut integrity [52,53]. An integrated analysis of cecal microbiota and serum metabolome in Guizhou yellow chickens showed that *R. torques* was enriched in high weight chickens and positively correlated with pantothenic acid (vitamin B5) [54]. We hypothesize the butyrate-producing function of the Lachnospiraceae family may play a role in gut health, and previous studies have tested the effects of butyrate supplementation in chickens infected with *E. maxima* [21,55]. Butyric acid glycerol esters can increase the abundance of Lactobacillaceae in *E. maxima* infected birds [21], and tributyrin supplementation can improve body weight gain and feed conversion ratio at the peak of infection (7 d PI) [55]. It remains a question if direct butyrate supplementation may differ from natural production from bacteria, however, it is possible supplementation also leads to increased natural butyrate production by increasing the relative abundance of Lachnospiraceae [56,57]. Multiple studies have found positive correlations between Lachnospiraceae abundance in the cecum with body weight gain, including in chickens infected by *E. tenella* [12], chickens challenged by a combination of *E. maxima* and *Clostridium perfringens* [20], and chickens supplied feed supplemented with fermented soybean meal [58].

The influence of taxa increased in IF birds on gut health is less clear. We observed increases to Lachnospiraceae *NK4A136 group*, *Merdibacter*, *Blautia*, *Colidextribacter*, unclassified Ruminococcaceae, and Clostridia *vadinBB60 group* in IF birds. Bacteria increased in CeM microbiota of IF birds were similar, with Lachnospiraceae *NK4A136 group*, *Merdibacter*, *Blautia*, unclassified Ruminococcaceae, and Clostridia *vadinBB60 group* also being increased in IF birds. The increase in two genera from the Lachnospiraceae family, *NK4A136 group* and *Blautia*, may be a response to infection, as these two genera have been correlated with differentially expressed genes related to inflammatory response in the chicken cecal microbiota [59]. This may explain why this pattern contradicts the usual decrease in Lachnospiraceae members in IF birds. Infection with *E. acervulina* has surprisingly shown the relative abundance of genera associated with beneficial functions can increase in cecal microbiota of IF birds [15], possibly because the microbiota responds differently to the more indirect effects of *E. acervulina* to the cecum compared to the physical damage that would occur from *E. tenella*. *E. tenella* infections have been characterized by major increases in potential secondary pathogens such as *Escherichia coli* in the cecal microbiota [10,12,60], which we did not observe in this study. Therefore, disturbance by *E. maxima* to the cecal microbiota may be more similar to that caused by *E. acervulina*, though the specific bacterial taxa increased in IF birds differ. One exception was *Merdibacter*, which was consistently increased in both CeL and CeM microbiota of IF birds in both studies [15].

Predictive functional analysis was utilized to investigate potential links between microbiota composition and metabolic pathways in IF and C birds. Outside of human studies, the accuracy of functional prediction can be limited [61], therefore, continued research using other methodology such as shotgun metagenomic sequencing, transcriptomics, and metabolomics are recommended. Our previous studies investigating effects of *E. tenella* and *E. acervulina* revealed predicted pathways related to butyrate production may be decreased in relative abundance in IF birds [12,13,15]. Included in those pathways were those which affect production of acetyl-CoA, an important molecule in two major pathways for butyrate production [47]. We again found IF birds were predicted to have decreased relative abundances in pathways related to acetyl-CoA production in the CeL microbiota of this study, including several pathways related to catechol degradation. In CeM microbiota, the succinate fermentation to butanoate and 4-aminobutanoate degradation V pathways were predicted to be decreased by infection, which could directly affect butyrate production. Changes in the abundances of these pathways may act as a link to understand why decreases in certain bacterial taxa correlate to decreased body weight gain in IF birds.

## Conclusions

*Eimeria maxima* infection affected alpha diversity in the CeL microbiota, decreasing Shannon diversity at d 3, increasing observed features at d 5, and increasing Shannon diversity at d 10 compared to C birds. In CeM microbiota, infection increased observed features at d 5, but later decreased observed features at d 14. CeL and CeM microbiota were not considered distinct between IF and C birds at each time point in beta diversity analysis, however, this could be further studied by understanding whether oocyst counts or time of year may influence this result. Consistent with studies on *E. acervulina* and *E. tenella*, the results suggest *Eimeria* infection may reduce relative abundance of butyrate-producing bacteria in the cecum, and abundance of pathways related to butyrate production are predicted to be reduced. Together, these studies show certain effects from *Eimeria* on the cecal microbiota are similar, despite differences in *Eimeria* species in their pathogenicity and the region of the GIT targeted. To expand beyond limitations of 16S sequencing, further research on the function of bacteria in the gut microbiota will assist in developing alternative solutions to antimicrobials such as probiotics or butyrate supplementation.
